# A Case of Posttransfusion Purpura with Severe Refractory Thrombocytopenia but No Cutaneous Manifestations

**DOI:** 10.1155/2018/8187659

**Published:** 2018-10-29

**Authors:** Jagjit Singh Bhamra, Per Ole Iversen, Thomas Kjenner Titze, Geir Erland Tjønnfjord, Çiğdem Akalın Akkök

**Affiliations:** ^1^Department of Immunology and Transfusion Medicine, Oslo University Hospital, Oslo, Norway; ^2^Department of Nutrition, Institute of Basic Medical Sciences, University of Oslo, Oslo, Norway; ^3^Department of Haematology, Oslo University Hospital, Oslo, Norway

## Abstract

Posttransfusion purpura is a serious adverse effect of transfusion due to HPA-antibodies. A young female was diagnosed with acute leukaemia, and treatment commenced. Severe thrombocytopenia ensued. No platelet increment was achieved despite transfusions with buffy coat, HLA-compatible, and HPA-1a negative platelets. The workup indicated the presence of anti-HPA-1a. When the diagnosis of posttransfusion purpura was sufficiently substantiated, she had experienced a fatal intracerebral haemorrhage.

## 1. Introduction

Posttransfusion purpura (PTP) is a rare transfusion reaction. In the British haemovigilance scheme, 56 PTP cases were reported in the period 1996–2015 [1]. PTP can occur following alloimmunisation to human platelet antigens (HPA), most commonly after pregnancies and/or blood transfusions. The antibody-mediated destruction of transfused platelets and the patients' own platelets can precipitate fatal bleedings. The preferred treatment is intravenous immunoglobulin (IVIg) [2].

### 1.1. Case Presentation

A young primipara was diagnosed with acute myeloid leukaemia (AML). She had haemoglobin (Hb) 9.9 g/dL (11.7–15.3), leukocytes 32.3 × 10^9^/L (3.5–11), platelets 119 × 10^9^/L (145–390), and D-dimer 1.3 mg/L (0.0–0.4). INR, APTT, and other blood values were normal.

**Figure 1:**
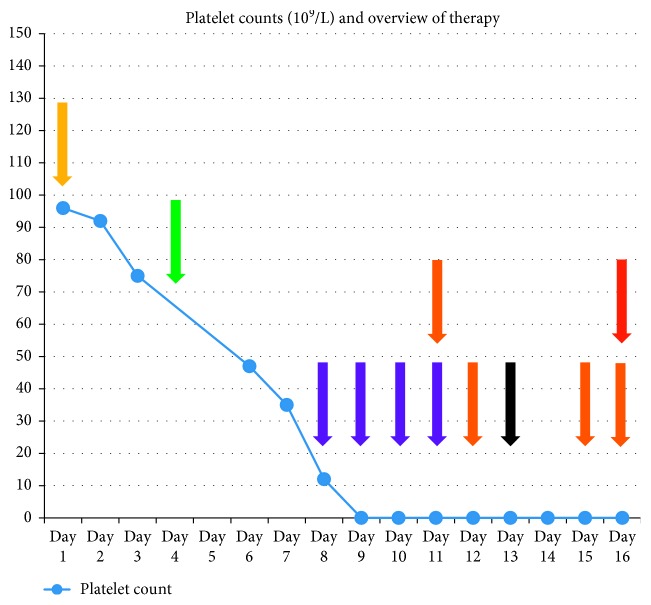
Platelet counts (10^9^/L) in our patient with AML, in relation to induction chemotherapy and all transfusions. Purple arrow indicates BCPC (buffy coat platelet concentrate): Day 8: BCPC × 2. Day 9: BCPC × 2. Day 10: BCPC × 1. Day 11: BCPC × 1. Orange arrow indicates HCPC (HLA-compatible platelet concentrates): Day 11: HCPC × 2. Day 12: HCPC × 1. Day 15: HCPC × 1. Day 16: HCPC × 4. Green arrow indicates PRBC (packed red blood cells): Day 4: PRBC × 2. Yellow arrow indicates Day 1: Start induction chemotherapy. Black arrow indicates Day 13: HPA‐1bb platelet concentrate x 1. Red arrow indicates Day 16: Death.

Induction chemotherapy started (Figure 1). On day 4, she received two units of leukoreduced packed red blood cells (PRBC) when Hb was 6.9 g/dL. On day 7, the induction chemotherapy was completed and antibiotics were initiated because of a lesion on her hand. The following night, she fainted and had haematochezia. Hb was 8.1 g/dL, and platelets were 12 × 10^9^/L; hence, one PRBC and one buffy coat platelet concentrate (BCPC) were given. Four more BCPCs were given on days 8-9 without any platelet increment. Luminex single-antigen assay revealed weak class I HLA-antibodies. Three HLA-compatible platelet concentrates (HCPCs) were transfused on days 10–12 but with no platelet increment. On day 13, one unit of HLA-compatible HPA-1bb platelet concentrate was transfused as HPA-antibodies were suspected, still without any platelet increment. Another HCPC was transfused on day 14 when she fell hitting her head.

On day 15, she presented with an acute stroke. Prothrombin complex concentrate, recombinant factor VIIa, four HCPCs, two PRBCs, and intravenous tranexamic acid were administered. A CT scan revealed an intracerebral haemorrhage (ICH) that was evacuated, but perioperative haemostasis was not achieved. She died of cerebral herniation.

A flow cytometric investigation on day 13 revealed increased reactivity to platelets from six HPA-1a positive donors and to lymphocytes from two of these donors. A crossmatch between the patient's plasma and platelets from an HPA-1bb donor was negative. These findings indicated the presence of anti-HPA-1a and a probable PTP.

## 2. Discussion

Our patient was transfused with two PRBCs, which probably triggered an anamnestic response by boosting anti-HPA-1a titres after a likely alloimmunisation during pregnancy. Four days after those transfusions, her platelet count was <10 × 10^9^/L, and it never increased despite repeated transfusions. The workup was consistent with the presence of anti-HPA-1a. Postmortem, she was genotyped to HPA-1bb and HLA-DRB3∗0101 positive, further supporting a PTP diagnosis.

Anti-HPAs can cause PTP and foetal neonatal alloimmune thrombocytopenia. Approximately 2% of Caucasians are positive for HPA-1bb [3]. Anti-HPA-1a made by them is the culprit antibody in 80–90% of PTP cases [3, 4]. PTP was first described by Shulman and coworkers [5]. The reported incidence is 1 : 50000–1 : 100000 [6], though PTP is likely underdiagnosed. The typical patient is a middle-aged, HPA-1bb female, who has been alloimmunised to HPA-1a in pregnancies and/or by blood transfusions. Male patients have been described [4]. Renewed exposure to the same antigen provokes an anamnestic response boosting alloantibody production. Other HPA-antibodies can also cause PTP [1, 3, 6].

Severe thrombocytopenia occurs 2–14 days [7] after the transfusion of a platelet-containing product (e.g., PRBCs, whole blood, platelet concentrates, and fresh plasma) with the foreign antigen [4, 6, 8]. Besides the transfused antigen-positive platelets, the recipient's antigen-negative platelets are also destroyed. Several mechanisms have been proposed to explain why: (1) Platelet antigen-positive blood transfusion triggers production of autoantibodies [9], (2) Transfused HPA-1a antigens are adsorbed onto the patient's own platelets [3], and (3) A cross-reaction between anti-HPA-1a and the patient's own platelets [10].

Symptoms include mucosal bleedings, haematomas, melena, haematuria, epistaxis, abnormal postoperative bleedings, and ICH. Not all have cutaneous manifestations [11]. Coagulation screens and bone marrow biopsies are usually normal [3], though obviously this would not be the case in our patient with AML. Untreated PTP lasts 7–28 days but can persist longer [3].

A thorough investigation can cause a diagnostic delay, which may warrant starting treatment before the workup is completed. Treatment with steroids and exchange transfusions [4] takes days and weeks before the thrombocytopenia resolves. About 80% of cases respond with a platelet increment within 48–72 hours following the administration of IVIg of 1–2 g/kg for two to five days or 500 mg/kg for five days [1–3]. The indication for platelet transfusions is controversial; however, the potential for preventing fatal bleedings may imply a liberal transfusion strategy.

Heparin-induced thrombocytopenia (HIT), disseminated intravascular coagulation (DIC), drug-induced thrombocytopenia (DIT), septicaemia, underlying haematological diseases, thrombotic microangiopathies, immune thrombocytopenia (ITP), and splenomegaly must be excluded [3]. Extraordinarily, passively transfused HPA-antibodies from a blood donor may cause a PTP-like condition in the recipient with shorter times for onset and spontaneous resolution [3]. A review of the medications our patient received excluded HIT and DIT. No findings were consistent with DIC, septicaemia, thrombotic microangiopathies, ITP, or splenomegaly. None of the abovementioned conditions have a temporal relationship with a preceding transfusion, unlike PTP.

HLA-antibodies can be present in PTP patients [3], necessitating further analyses to identify the culprit alloantibody. Monoclonal antibody-specific immobilisation of platelet antigen assay (MAIPA) or Luminex-based assay is used to resolve the presence and specificity of the HPA-antibody. HLA-DRB3∗0101 typing should be performed as this tissue type is assumed to play an HPA-1a antigen-presenting role [12] and HLA-DRB3∗0101 individuals are prone to producing anti-HPA-1a. A drawback of our investigation was that MAIPA was not performed due to lack of a suitable sample.

## 3. Conclusion

Not all PTP patients have purpura justifying the name of the condition, and the lack of this may lead to diagnostic and therapeutic delays. However, refractoriness to all kinds of platelet transfusions is the typical finding. We, therefore, suggest this condition should be called posttransfusion refractory thrombocytopenia (PTRT).
